# Urine biomarkers in type 2 diabetes mellitus with or without microvascular complications

**DOI:** 10.1038/s41387-024-00310-5

**Published:** 2024-07-10

**Authors:** Chanyuan Zhang, Tiebing Liu, Xiaoqian Wang, Jing Yang, Dongfang Qin, Yin Liang, Xuejing Wang

**Affiliations:** 1 Department of Clinical Laboratory, Civil Aviation General Hospital (Peking University Civil Aviation School of Clinical Medicine), Beijing, China; 2 Civil Aviation Medicine Center, Civil Aviation Administration of China (Civil Aviation General Hospital), Beijing, China

**Keywords:** Diabetes, Proteins

## Abstract

**Objective:**

To investigate the distribution of nine (9) urine biomarkers in people living with type 2 diabetes mellitus (T2DM), with or without microvascular complications.

**Methods:**

In total, 407 people with T2DM were enrolled from 2021 to 2022. According to diabetic retinopathy (DR) and urinary albumin-creatinine ratio (UACR), the 407 people were divided into four (4) groups, DR(–)UACR(–), DR(+)UACR(–), DR(–)UACR(+), and DR( + )UACR(+). In addition, 112 healthy volunteers were enrolled during the same period. The nine (9) urine markers included α1-microglobulin (u-α1MG), immunoglobulin G (u-IgG), neutrophil gelatinase-associated lipid carrier protein (u-NGAL), cystatin C (u-CysC), retinol-binding protein (u-RBP), β2-microglobulin (u-β2MG), N-acetyl-β-D-glucosaminidase (u-NAG), transferrin (u-Trf), and collagen type IV (u-Col). For each marker, the respective level of 97.5 percentile in healthy volunteers was taken as an upper reference limit.

**Results:**

Among the 407 people, 248 individuals (61%) were DR(–)UACR(–), 100 (25%) were DR(-)UACR(+), 37 (9%) were DR(+)UACR(–), and 22 (5%) were DR(+)UACR(+). The u-NAG/Cr biomarker level showed a significant difference between healthy participants and people with T2DM. In the DR(–)UACR(–)group, u-Trf/Cr showed the highest positive rate (21.37%), followed by u-IgG/Cr (14.52%); u-NAG/Cr (10.48%); u-β2MG/Cr (4.44%); u-CysC/Cr (4.03%); u-NGAL/Cr (4.03%); u-RBP/Cr (2.82%); u-α1MG/Cr (2.42%); 17.34% of people with T2DM showed multiple biomarkers positive (≥2 biomarkers). The positive rates of one biomarker (21.33%) and two biomarkers (18.67%) in people who have less than five (5) years of T2DM were almost close to those of the DR(–)UACR(–) group (21.37%, and 12.10%, respectively).

**Conclusion:**

Renal tubule biomarkers may be used as an indicator in the early detection and monitoring of renal injury in diabetes mellitus. The u-NAG biomarker should be measured for the people with T2DM of the first-time diagnosis.

## Introduction

Diabetic kidney disease (DKD) is one of the most common microvascular complications of diabetes mellitus (DM). The prevalence of DKD is reported to be about 20–40% worldwide with an increasing trend [1]. As it is deemed the main cause of end-stage renal disease (ESRD) and increases the risk of death [2, 3], early detection and intervention are crucial [4, 5]. Although renal biopsy is often considered the gold diagnostic standard, it is also very invasive [6]. Urinary albumin-creatinine ratio (UACR) plays an important role in the early screening of DKD. However, studies proved that even in DM patients with normoalbuminuria, there also was a progressive decrease in eGFR [7]. Diabetic retinopathy (DR) is another microvascular complication of DM and is considered an important sign of diabetic kidney disease [8]. At the same time, some people living with DKD are not accompanied by DR [9]. There is also increasing attention on renal tubulointerstitial injury, which may occur earlier than glomerular damage [5, 10, 11]. In this study, we assessed the detectability and distribution of 9 urine biomarkers in people living with type 2 diabetes mellitus (T2DM), with or without microvascular complications, aiming to discover their added utility in the earlier detection of kidney damage in addition to DR and UACR.

## Methods

### Study participants

Between February 2021 and December 2022, 407 people with T2DM were enrolled by continuous and convenient sample collection at the Endocrinology Department of Civil Aviation General Hospital, Beijing, China. Inclusion criteria are (1) satisfaction with the WHO diagnostic criteria for diabetes in 2021 [12], (2) 18–80 years old, and (3) completion of the examination of the retina. Exclusion criteria are any known acute and chronic kidney diseases, allergies, connective tissue diseases, infections, tumors, ketoacidosis, and heart failure. At the same time, 112 healthy subjects aged 18 to 80 years from the physical examination department were also enrolled to establish a normal range of any urine biomarkers. This study was conducted in accordance with the principles of the Declaration of Helsinki and was approved by the Ethics Committee of the Civil Aviation General Hospital (2022-L-K-53). Informed consent was obtained from all subjects.

### Materials and methods

DR was evaluated by an ophthalmologist based on the slit lamp examination and fundus photograph of the retina, according to reported guidelines [13, 14]. Urine samples of participants were collected on the second morning after fasting. All urine samples were analyzed with an automatic biochemical analyzer (Hitachi P modular). The urine markers included: α1-Microglobulin (u-α1MG), immunoglobulin G (u-IgG), microalbumin (u-Alb), neutrophil gelatinase-associated lipid carrier protein (u-NGAL), cysteine protease inhibitor C (u-CysC), retinol-binding protein (u-RBP), β2-Microglobulin (u-β2MG), N-acetyl-β-D-glucosaminidase (u-NAG), transferrin (u-Trf), collagen type IV (u-Col), and urine creatinine (Cr).

It has been recognized that these biomarkers are associated with certain functions of a kidney. For example, UACR, u-Trf/Cr, and u-IgG/Cr are deemed markers of glomerular barriers. U-α1 MG/Cr, u-β2 MG/Cr, u-RBP/Cr, and u-CysC/Cr are deemed as markers of renal tubular reabsorption. U-NGAL/Cr and u-NAG/Cr are deemed markers of renal tubular epithelial cell injury. U-Col/Cr is deemed as a marker of glomerular and tubular basement membrane. As DKD can damage glomerular barriers, the renal tubular reabsorption, renal tubular cells, and the membrane, the above-listed biomarkers can show any abnormal situation of these functions, which may serve as an early indicator of DKD.

The biomarker assays were supplied by Beijing Leadman Biochemistry Co. Ltd, and the calibration of the testing kits has been reported in another publication [15]. The performance of the 9 urine biomarkers assays was presented in Table 1. After centrifugation, the supernatant of urine was analyzed, and all the concentrations were calibrated by urine creatinine.

**Table 1:** Performance of nine urine biomarker assays.

Biomarker	Test method	Precision CV (%)		Linear range
α1-microglobulin (mg/L)	Latex immunoturbidimetry	Low value	2.15	0.83–28.50
		High value	1.19	
β2-microglobulin (mg/L)	Immunoturbidimetry	Low value	3.00	0.06–4.40
		High value	1.16	
Immunoglobulin G (mg/L)	Immunoturbidimetry	Low value	4.01	4.27–160.10
		High value	1.59	
N-Acetyl-β-D-glucosaminidase (U/L)	MPT method	Low value	5.73	0.90–220.10
		High value	1.51	
Neutrophil gelatinase-associated lipocalin (µg/L)	Latex immunoturbidimetry	Low value	4.49	5.33–4846.00
		High value	2.72	
Transferrin (mg/L)	Immunoturbidimetry	Low value	4.78	1.90–768.66
		High value	2.55	
Urine creatinine (µmol/L)	Sarcosine oxidase method	Low value	1.20	300.00–60000.00
		High value	1.10	
Cystatin C (mg/L)	Latex enhanced immunoturbidimetry	Low value	5.37	0.10–8.91
		High value	1.66	
Retinol binding protein (mg/L)	Latex immunoturbidimetry	Low value	4.68	0.33–30.81
		High value	3.59	

### Statistical analysis

As all variables do not follow the normal distribution, a Mann-Whitney, or Kruskal-Wallis rank sum test was used to compare groups, and a pairwise comparison was conducted with the Benjamini-Hochberg method. Categorical variables were expressed in terms of the number of subjects and their corresponding percentages, and an χ2 test or exact probability method was used for comparisons between groups. Statistical significance was defined as *p* < 0.05. The data analysis was conducted using Stata 12.0 and R software.

## Results

### Distribution of nine urine biomarkers in healthy individuals

In the 112 healthy individuals, the nine (9) urine biomarkers showed skewed distributions. The quantiles at the 2.5th, 25th, 50th, 75th, and 97.5th percentiles are presented in Table 2. The level of 97.5 percentile was taken as the upper reference limit for each biomarker. It was considered positive if the concentration was above the upper reference limit. For UACR, the cut-off value was set at >30 mg/g [16].

**Table 2:** Concentration distribution of nine urinary biomarkers.

Biomarker	2.5th percentile	25th percentile	50th percentile	75th percentile	97.5th percentile
u-α 1 MG/Cr (mg/g)	0.44	1.18	2.22	3.49	8.46
u-β 2 MG/Cr (mg/g)	0.00	0.04	0.12	0.24	1.01
u-IgG/Cr (mg/g)	0.00	0.00	0.00	0.61	3.82
u-Col/Cr (mg/g)	0.00	0.97	3.05	6.83	36.72
u-NAG/Cr (U/g)	0.00	1.19	2.15	3.34	9.97
u-NGAL/Cr (mg/g)	0.00	0.00	0.02	0.05	0.18
u-Trf /Cr (mg/g)	0.00	0.00	0.04	0.29	1.61
u-RBP/Cr (mg/g)	0.00	0.00	0.00	0.01	0.15
u-CysC/Cr (mg/g)	0.00	0.02	0.03	0.05	0.13

*u-α1MG* α1 microglobulin, *u-IgG* imunoglobulin G, *u-Ab* microalbumin, *u-NGAL* ueutrophil gelatinase-associated lipid carrier protein, *u-CysC* cystatin C, *u-RBP* retinol-binding protein, *u-β2MG* β2 microglobulin, *u-NAG* N-acetyl-β-D-glucosaminidase, *u-Trf* transferrin, *u-Col* u-Collagen type IV, *Cr* urine creatinine.

### The proportion of microvascular complications in 407 people with T2DM

**Fig. 1:**
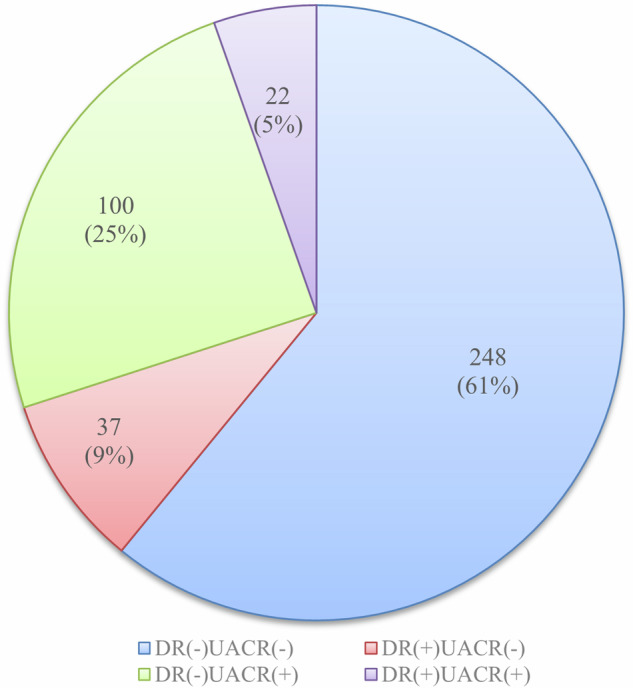
The proportion of microvascular complications in 407 people with T2DM. In the pie chart, the blue area represents the DR (-) UACR (-) group, the red area represents the DR (+) UACR (–) group, the green area represents the DR (–) UACR (+) group, and the purple area represents the DR (+) UACR (+) group.

Among the 407 people with T2DM, 248 individuals (61%) were in the group of DR(-)UACR(-), 100 individuals (25%) were in the group of DR(–)UACR( + ), 37 individuals (9%) were in the group of DR( + )UACR(–), and 22 individuals (5%) were in the group of DR( + )UACR(+). Figure 1 illustrates a pie chart showing the distribution of people with T2DM. The clinical characteristics of the enrolled population are shown in Supplementary Tables 1, 2.

### Comparison of urine biomarkers among different groups

#### Distribution of urinary biomarkers among different groups

**Fig. 2:**
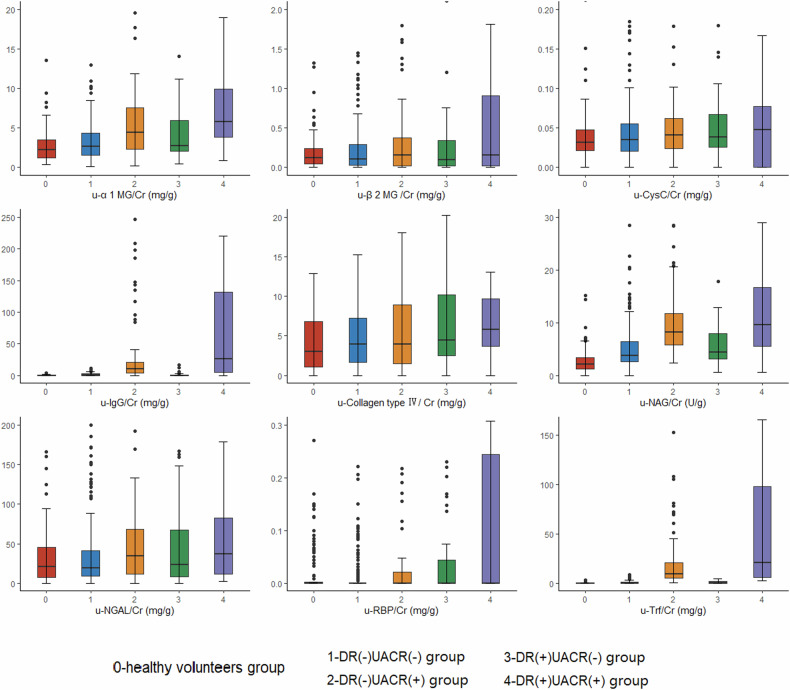
Distribution of the urinary biomarkers in healthy individuals and people with T2DM. Each graph includes levels of a biomarker corresponding to five (5) groups, where 0 represents the healthy participants, and 1–4 represent DR(–)UACR(–), DR(–)UACR( + ), DR( + )UACR(–), and DR( + )UACR(+) group, respectively.

Figure 2 shows the levels of glomerular and tubular injury biomarkers in each group and according to different microvascular complications. The DR( + )UACR(+) group has a much higher level than any other group with regard to every biomarker. Compared with the levels of healthy volunteers, the DR(-)UACR(-) group showed a significantly higher level for u-NAG/Cr, indicating that u-NAG is an earlier marker for tubular damage. Besides, the UACR(-) group showed an increasing trend for u-α1 MG/Cr and u-NAG/Cr, with or without retinopathy. For the DR(+) group, u-RBP/Cr showed a higher level than those of the DR(-) group with the same level of UACR, which might be a potential marker for retinopathy.

#### The positive rates of the urine biomarkers among different groups

Table 3 shows the positive rates of the nine (9) urine biomarkers among different groups. The positive rates of eight (8) urine biomarkers were significantly higher in the UACR(+) group than those of the UACR(–) group, except for u-NGAL/Cr among DR(–) subjects. Among DR(+) subjects, only the detection rates of u-Trf/Cr, u-IgG/Cr, and u-NAG/Cr were significantly higher in the UACR(+) group than in the UACR(–) group. Among the DR(–)UACR(–) group, the u-Trf/Cr showed the highest positive rate (21.37%), followed by u-IgG/Cr (14.52%); u-NAG/Cr (10.48%); u-β2MG/Cr (4.44%); u-CysC/Cr (4.03%); u-NGAL/Cr (4.03%); u-RBP/Cr (2.82%); u-α1MG/Cr (2.42%); and u-Col/Cr (0.00%). Except for u-β2MG/Cr, u-RBP/Cr, and u-CysC/Cr, there were no significant differences among the three groups in 189 subjects living with T2DM (Supplementary Table 3). The positive rate of u-NAG/Cr was 11.27% in the non-hypertension group among the DR(-)UACR(-) group (Supplementary Table 4).

**Table 3:** The detection rate of nine urinary biomarkers in people with T2DM among different groups.

Biomarkers	DR(-)UACR(-)	DR(-)UACR(+)	DR( + )UACR(-)	DR( + )UACR(+)
	(*N* = 248)	(*N* = 100)	(*N* = 37)	(*N* = 22)
**Glomerular barrier markers**				
u-Trf/Cr	53 (21.37%)	97 (97.00%)	10 (27.03%)	22 (100.00%)
u-IgG/Cr	36 (14.52%)	73 (73.00%)	3 (8.11%)	17 (77.27%)
**Markers of renal tubular reabsorption**				
u-α 1 MG/Cr	6 (2.42%)	20 (20.00%)	5 (13.51%)	7 (31.82%)
u-β 2 MG/Cr	11 (4.44%)	13 (13.00%)	4 (10.81%)	5 (22.73%)
u-RBP/Cr	7 (2.82%)	19 (19.00%)	6 (16.22%)	6 (27.27%)
u-CysC/Cr	10 (4.03%)	11 (11.00%)	3 (8.11%)	4 (18.18%)
**Markers of renal tubular epithelial cell injury**				
u-NGAL/Cr	10 (4.03%)	8 (8.00%)	1 (2.70%)	1 (4.55%)
u-NAG/Cr	26 (10.48%)	35 (35.00%)	5 (13.51%)	11 (50.00%)
**Glomerular and tubular basement membrane markers**				
u-Col/Cr	0 (0.00%)	3 (3.00%)	1 (2.70%)	2 (9.09%)

*DR* diabetes retinopathy, *UACR* albumin-to-creatinine, *u-α1MG* α1 microglobulin, *u-IgG* immunoglobulin G, *u-Ab* microalbumin, *u-NGAL* neutrophil gelatinase-associated lipid carrier protein, *u-CysC* cystatin C, *u-RBP* retinol-binding protein, *u-β2MG* β2 microglobulin, *u-NAG* N-acetyl-β-D-glucosaminidase, *u-Trf* transferrin, *u-Col* u-Collagen type IV, *Cr* urine creatinine.

#### Multiple positive rates of urine biomarkers in T2DM patients

**Fig. 3:**
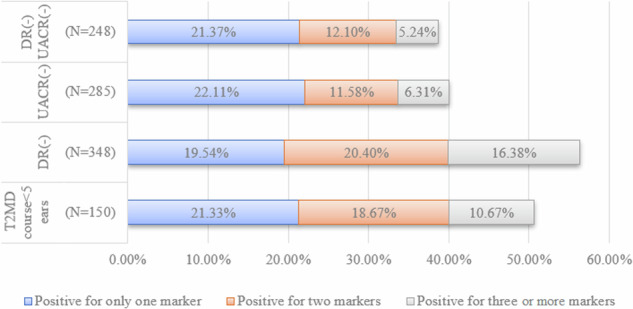
Multiple positive rates of urine biomarkers in 407 people with T2DM. In the bar chart, the blue area represents the positive rate of only one marker, the red area represents the positive rate of both two markers, and the gray area represents the positive rate of simultaneous three or more markers.

Among people of the DR(–)UACR(–) group, 21.37% were positive for only one marker, and 17.34% showed multiple positives (≥2 biomarkers). Among the individuals with a diabetes duration of less than 5 years, 21.33% were positive for only one urine biomarker. The top three urine biomarkers that have high positive rates were u-Trf/Cr, u-NAG/Cr, and u-IgG/Cr. The DR(–)UACR(–) group surprisingly showed the same three urine biomarkers with high positive rates. (Fig. 3, Supplementary Fig. 1).

## Discussion

The first finding of our research was the distribution of microvascular complications in diabetes in the outpatient population. Among the enrolled outpatient population, about 61% of the people fall into the group DR(–)UACR(–), 9% fall into the group DR( + )UACR(–), 25% fall into the group DR(–)UACR(+), and 5% fall into the group of DR( + )UACR(+). In these outpatient populations, about 14% of DR(+) people showed significant differences in the measured value or positive rate of urine markers regardless of the development of retinopathy, suggesting that the occurrence of retinopathy and kidney damage in people with diabetes may not be closely associated with each other at least in certain patients. While some studies pointed out that DR and DKD might have been accompanied by and influenced each other [17, 18], our study showed a sizable portion of people with T2DM may develop the two conditions independently from each other.

Our second finding was that even in the patients having both negative values in DR and UACR, the positive detection rate of u-NAG/Cr reached 10.48%. It has been recognized that urinary NAG, which is located on the lysosome of renal tubular epithelial cells, can reflect the structural damage of renal tubular epithelial cells [19]. People with T2DM often have an elevated level of u-NAG/Cr possibly due to the overload burden of re-absorption, the local inflammation injury and hypoxia, etc. These conditions might happen before the glomerular damage. A previous study also showed that the u-NAG level was proportional to non-albumin proteinuria levels in T2DM patients with DKD even before the onset of overt albuminuria [20]. In the UACR positive group, the u-NAG/Cr positive rate was close to 40%, indicating that the effect of diabetes on the glomeruli and tubules was independent, rather than in concert, and varied from person to person. This finding strongly suggests that it is important to include renal tubular-related markers in the early screening of DKD. Further studies about the susceptibility of people with T2DM to glomerular or tubular injury are needed, and appropriate early intervention strategies also need further investigation.

Our third finding was that the positive rates of u-IgG/Cr were high even in the people with both negative values in DR and UACR. Previous research suggests that the increase in urinary IgG reflects the serious damage to the glomerular basement membrane [21]. The increase in a urinary IgG excretion rate seems to suggest a decrease in the estimated glomerular filtration rate (eGFR), which may be a marker of disease progression [22]. However, some other studies have reported that urinary IgG can rise before the occurrence of microalbuminuria or in normal albuminuria in people with diabetes [23–25], which is consistent with our results. It is possible that the increase in urinary IgG excretion may be due to selective damage to the glomerular pore size and increased intraglomerular water pressure caused by hyperglycemia [26]. As urine IgG has a higher sensitivity than that of microalbuminuria in reflecting changes in renal hemodynamics and inflammation [27], our finding suggests that urinary IgG can also be used as an early marker of diabetic kidney injury.

Our study also showed several abnormal increases in other urine proteins, including both glomerular and tubular relative biomarkers. The data shows that urinary renal tubular reabsorption biomarkers (such as u-α1MG/Cr, u-β2MG/Cr, u-RBP/Cr, and u-CysC/Cr) increased in the DR and UACR double negative group. This result suggests that the proximal tubule reabsorption function may be decreased in the early stage of diabetes, which can cause a large amount of low molecular weight proteins to be present in the urine [7]. The detection rates of tubular epithelial cell injury biomarkers (u-NGAL/Cr and u-NAG/Cr) were 4.03% and 10.48% respectively. The development of inflammation, stress, and increased reabsorption burden caused by high glucose could worsen renal tubular lesions. These biomarkers could help to evaluate the extent of renal tubular injury [28, 29]. The management and prognosis based on these biomarkers could be clinically significant and are worthy of further investigation.

We finally found that the positive rates for certain biomarkers did not show significant differences in patients having different years of diabetes. In both the group of diabetes with less than 5 years and other groups, it is observed that the top three biomarkers that have high positive rates were u-TRF/Cr, u-NAG/Cr, and u-IgG/Cr. This is true even in the DR(-)UACR (-) group. (See Fig. 3, Supplementary Fig. 1) As the onset of type 2 diabetes is difficult to pinpoint precisely, it is advisable to initiate annual testing for albuminuria and tubular injury markers at the time of diabetes diagnosis [16]. However, tubular markers might not have been frequently measured in the present clinical work, causing their clinical significance to be underappreciated to certain degrees. We suggest that different panels of tubular biomarkers should be used for diagnosing people living with T2DM at different diabetes duration for various purposes, to evaluate DKD more completely.

The study has several limitations. Firstly, it was a cross-sectional study that adopted continuous and convenient recruitment, and we provided the distribution of nine (9) urinary biomarkers only in people living with T2DM in a stable chronic condition. Secondly, due to limited conditions, we did not collect detailed medication information of the participants, such as their medication history. The eGFR and HbA1c results were collected for only a small number of participants. As a result, we were unable to thoroughly discuss any correlation among eGRF, HbA1c, and the results of nine (9) biomarkers in this study. Thirdly, we didn’t determine the range of the sample size for this study. When planning this study, we set up a minimum threshold and then tried to enroll as many participants as possible within the period. In addition, this is a single-center study.

In summary, diabetes retinopathy and renal lesions can be independent, and even a seemingly normal UACR could not rule out retinopathy. It is necessary to add renal tubule markers to the screening list of DKD, to fill in the gap in UACR-negative people living with T2DM. Early detection of renal tubular biomarkers is critical for adjusting treatment strategies, safeguarding kidney functions, and improving prognosis. Detection of both glomerular and tubule biomarkers is recommended in efforts to detect DKD in the early stages.

## Supplementary information

Supplementary information

Supplementary Fig 1. Positive counts for only one of the nine urinary biomarkers in four groups. A represents diabetes duration 10 years group, B represents diabetes duration 5-10 years group, C represents diabetes duration > 10 years group, and D represents DR(-)UACR (-) group.

Supplementary Table 1. Clinical characteristics of 407 people with T2DM among different groups

Supplementary Table 2. Clinical characteristics and urinary biomarkers of 407 people with T2DM by UACR groups

Supplementary Table 3. Clinical characteristics and urinary biomarkers of 189 people with T2DM by eGFR groups

Supplementary Table 4. Clinical characteristics and urinary biomarkers of 203 people with T2DM but without hypertension

Supplementary Table 5. Clinical characteristics and urinary biomarkers of 204 people with T2DM and hypertension

## Data Availability

All data included in this study are shown in this article or supplementary information, any further request is available by contacting the corresponding authors.
