# Supplementary material for: Urine biomarkers in type 2 diabetes mellitus with or without microvascular complications
Source: Nutr Diabetes. 2024 Jul 10;14:51. doi: 10.1038/s41387-024-00310-5 (PMC11236963; doi:10.1038/s41387-024-00310-5)
Supplement: Supplementary file 1 — Supplementary information [file 41387_2024_310_MOESM1_ESM.docx]

**Supplementary figure and table legends**

**Supplementary Fig 1. Positive counts for only one of the nine urinary biomarkers in four groups.** A represents diabetes duration 10 years group, B represents diabetes duration 5-10 years group, C represents diabetes duration > 10 years group, and D represents DR(-)UACR (-) group.

**Supplementary Table 1. Clinical characteristics of 407 people with T2DM among different groups.**

**Supplementary Table 2. Clinical characteristics and urinary biomarkers of 407 people with T2DM by UACR groups.**

**Supplementary Table 3. Clinical characteristics and urinary biomarkers of 189 people with T2DM by eGFR groups.**

**Supplementary Table 4. Clinical characteristics and urinary biomarkers of 203 people with T2DM without hypertension.**

**Supplementary Table 5. Clinical characteristics and urinary biomarkers of 204 people with T2DM and hypertension.**
